# The tales of two cities: use of evidence for introducing 20 miles per hour speed limits in Edinburgh and Belfast (United Kingdom)

**DOI:** 10.1186/s12961-024-01213-8

**Published:** 2024-09-02

**Authors:** Karen Milton, Graham Baker, Claire L. Cleland, Andy Cope, Ruth F. Hunter, Ruth Jepson, Frank Kee, Paul Kelly, Andrew J. Williams, Michael P. Kelly

**Affiliations:** 1 Norwich Medical School, University of East Anglia, Norwich, Norfolk NR4 7TJ United Kingdom; 2 Physical Activity for Health Research Centre (PAHRC), Institute for Sport, Physical Education and Health Sciences, University of Edinburgh, Edinburgh, United Kingdom; 3 School of Medicine, Dentistry, and Biomedical Sciences, Queen’s University, Belfast, United Kingdom; 4 Sustrans, United Kingdom; 5 Centre for Public Health, Queen’s University, Belfast, United Kingdom; 6 Scottish Collaboration for Public Health Research and Policy, University of Edinburgh, Edinburgh, United Kingdom; 7 Division of Population and Behavioural Science, School of Medicine, University of St Andrews, St Andrews, United Kingdom; 8 Primary Care Unit, Department of Public Health and Primary Care, University of Cambridge, Cambridge, United Kingdom

**Keywords:** 20 mph, Speed limit, Evidence, Policy

## Abstract

**Background:**

In 2016, large-scale 20 miles per hour speed limits were introduced in the United Kingdom cities of Edinburgh and Belfast. This paper investigates the role that scientific evidence played in the policy decisions to implement lower speed limits in the two cities.

**Methods:**

Using a qualitative case study design, we undertook content analysis of a range of documents to explore and describe the evolution of the two schemes and the ways in which evidence informed decision-making. In total, we identified 16 documents for Edinburgh, published between 2006 and 2016, and 19 documents for Belfast, published between 2002 and 2016.

**Findings:**

In both cities, evidence on speed, collisions and casualties was important for initiating discussions on large-scale 20 mph policies. However, the narrative shifted over time to the idea that 20 mph would contribute to a wider range of aspirations, none of which were firmly grounded in evidence, but may have helped to neutralize opposing discourses.

**Discussion and conclusions:**

The relationship between evidence and decision-making in Edinburgh and Belfast was neither simple nor linear. Widening of the narrative appears to have helped to frame the idea in such a way that it had broad acceptability, without which there would have been no implementation, and probably a lot more push back from vested interests and communities than there was.

## Introduction

In transport policy in the United Kingdom (UK), there has been a long-standing interest in the development and use of evidence. In 1933, the Road Research Laboratory was established and many of the policies that have improved vehicle and pedestrian safety over the years in the UK are a consequence of the engineering–scientific approach of the old Ministry of Transport and that continued by the Department for Transport [6]. The idea that the best scientific evidence should be a major part of policy decision-making processes gained considerable traction in the last several decades in arenas other than transport, including the widespread promotion and adoption of evidence-based medicine [39, 40, 48] and subsequently evidence-based public health [2].

Even if the acceptance by decision-makers of scientific evidence is sometimes more rhetorical than real, the notion that evidence is important in decision-making is widely acknowledged [17, 24, 26, 38]. There is a recognized need for transparency in the policy decision-making process, such that the public, as well as experts and officials, can understand the science and motivation behind a policy [45]. However, the policy-making process involves an extremely complex “web” of individuals, organizations, events and decisions. As such, many factors other than evidence play into the decision-making processes, including public opinion, interest groups and the economic climate, to name but a few [3].

The complexity of policy-making is exemplified by the range of theories that aim to simplify the process so that it can be more easily understood (see [5]). Some commentators have talked about “policy narratives”, which have been defined as currents that sit above policies, acting as a rallying call to those across government and between government and non-government entities, providing directional pointers and broad benchmarks for change [15]. The analysis of policy narratives is concerned with the way in which policy actors frame issues to focus the attention of their audience and to shape the way they interpret information.

A transport policy trialled in several UK cities over the past 10–15 years is 20 miles per hour (mph) speed restrictions, which have typically taken two forms. More traditionally used are 20 mph “zones” which involve the installation of physical infrastructure such as speed bumps or chicanes. The other approach is 20 mph “limits”, which involve the installation of “signs and/or lines” without any other physical traffic calming infrastructure [28]. While there is evidence that zones are effective in reducing collisions and casualties, prior to the introduction of 20 mph limits in Edinburgh and Belfast, rather less was known about their effectiveness [13]. Nonetheless, decisions were made in the two UK cities of Edinburgh and Belfast to implement large-scale 20 mph limits in 2016.

Our previous research has highlighted that in both Edinburgh and Belfast the policy decisions to enact 20 mph speed limits followed many years of discussion and deliberation [29]. While a range of factors have been identified as being important in decision-making processes locally, for example, a favourable national policy context, political leadership and public support [29], one key issue remains unexplored – the role of evidence itself. Therefore, the aim of this paper was to investigate the policy narratives in the two cities and the role that evidence played (or not) in the processes that led to the introduction of 20 mph speed limits in the two cities.

## Methods

### Study design

Due to the complex contextual factors influencing the decisions to introduce 20 mph speed limits, a qualitative case study design was deemed appropriate [56]. The cases were defined as the policies to introduce 20 mph speed limits in each city and were bounded in time, dating back to the earliest documented discussions about the schemes, up to the decisions to proceed with implementation in 2016.

### Data sources and collection

The data reported here were gathered as part of a broader National Institute for Health Research (NIHR)-funded project examining the processes and outcomes of the introduction of 20 mph speed limit interventions in the cities of Edinburgh and Belfast [25].

The data were collected from documents available in the grey literature. We conducted searches of relevant websites to identify papers about 20 mph interventions, including UK-wide and Scottish and Northern Irish developments. The websites included those of the national governments (Scotland and Northern Ireland) and the City of Edinburgh Council – the local authority responsible for services such as education, housing and waste disposal in Edinburgh. We did not look for local council documents in Belfast, as the initiative there was led and managed by the Northern Ireland Government – the Department for Regional Development, which changed its name to the Department for Infrastructure in May 2016. We searched the websites for any documents related to transport policy and road safety. We sought to identify legislation, policy statements, responses to public consultations, research reports and official statistics, as well as other written records of events including official announcements, committee reports, and debates. Search terms included “20mph”, “speed limits”, “speed restrictions” and “road safety”. No limit was set on publication date.

Grey literature can be difficult to search and retrieve [1, 30], and we found this to be the case, particularly for Belfast. As such, a member of the research team (RFH) worked closely with the Department for Infrastructure in Northern Ireland to determine what documents existed and how the research team could gain access to them. Building good relationships was important in subsequently obtaining relevant documentation for Belfast.

We compiled a timeline of the publication of relevant documents for each of the two cities, respectively. These were shared with a range of stakeholders in both places, including members of the NIHR study steering committee and people who took part in interviews as part of the broader project [25]. These stakeholders were asked to confirm the accuracy and comprehensiveness of the list of documents in the timelines. Any additional documents identified by the stakeholders were located and included in the analysis. In total, we identified 16 documents for Edinburgh, published between 2006 and 2016, and 19 documents for Belfast, published between 2002 and 2016 (See Tables 1 and 2 for a chronological list of documents for each city). We obtained electronic copies of all documents for inclusion in the analysis.

**Table 1:** Edinburgh documents related to 20 mph (*n* = 16)

Date (month, year)	Title	Author
November 2006	Edinburgh’s Local Transport Strategy	City of Edinburgh Council
June 2009	Scotland’s Road Safety Framework to 2020	Scottish Government
March 2010	Transport 2030 Vision	City of Edinburgh Council
May 2010	Road Safety Plan for Edinburgh to 2020	City of Edinburgh Council
September 2010	Proposal for 20 mph Speed Limit Pilot in South Edinburgh	City of Edinburgh Council
September 2010	Relationship between Speed and Risk of Fatal Injury: Pedestrians and Car Occupants	UK Department for Transport
November 2010	Active Travel Action Plan	City of Edinburgh Council
February 2011	20 mph Speed Limit Pilot in South Edinburgh	City of Edinburgh Council
August 2011	South Edinburgh 20 mph Limit Pilot – Response to TRO Consultation	City of Edinburgh Council
Nov, 2011	20 mph Speed Limit Pilot in South Edinburgh – Variation to Traffic Regulation Order	City of Edinburgh Council
August 2013	South Central Edinburgh 20 mph Limit Pilot Evaluation	City of Edinburgh Council
January 2014	Local Transport Strategy 2014–2019	City of Edinburgh Council
January 2014	Delivering the Local Transport Strategy 2014–2019: 20 mph Speed Limit Roll Out – Proposed Network	City of Edinburgh Council
December 2014	Good Practice Guide on 20 mph Speed Restrictions	Transport Scotland
March 2015	20 for Edinburgh: 20 mph Network Implementation	City of Edinburgh Council
January 2016	Objections to Traffic Regulation Order TRO/15/17 20 mph Speed Limit – Various Roads, Edinburgh	City of Edinburgh Council

**Table 2:** Belfast documents related to 20 mph (*n* = 19)

Date (month, year)	Title	Author
July 2002	Northern Ireland Road Safety Strategy 2002–2012	NI Government
July 2008	20 mph speed limit signs at schools	NI Government
February 2010	20 mph Part-Time Speed Limits: Report on Pilot Studies	NI Government
April 2010	Setting Local Speed Limits in Northern Ireland	NI Government
March 2011	Northern Ireland’s Road Safety Strategy to 2020	NI Government
February 2014	An Overview of Key Road Traffic Collisions Statistics in Northern Ireland	Des McKibbin, Research and Information Service. Briefing paper for Northern Ireland Assembly
February 2014	Road Traffic (Speed Limits) Bill	Des McKibbin, Research and Information Service. Briefing paper for Northern Ireland Assembly
April 2014	Official Report for the Northern Ireland Assembly	Danny Kennedy, Committee for Regional Development, NI Government
June 2014	Road Safety Engineering Procedures	Transport NI
June 2014	Road Safety at Schools	Transport NI
August 2014	Proposed 20 mph speed limit in Belfast City Centre	Belfast City Council
August 2014	Table of objections in response to Proposed 20 mph speed limit in Belfast City Centre	Belfast City Council (not publicly available)
February 2015	Pilot 20 mph Schemes in Belfast City Centre, Merville Garden Village and Ballymena (revision)	Transport NI
February 2015	Meeting to discuss Proposed 20 mph Speed Limit in Belfast City Centre	Transport NI (not publicly available)
March 2015	Objections to the Proposed 20 mph Speed Limit in Belfast City Centre	Transport NI (not publicly available)
September 2015	The Roads (Speed Limit) (No. 3) Order (Northern Ireland) 2015	NI Government
October 2015	Minutes from Committee Meeting 14th October 2015, in respect to a request for additional information on 20 mph following the publication of the Roads (Speed Limit) (No. 3) Order (Northern Ireland) 2015	NI Government (not publicly available)
November 2015	20 mph Signed Only Speed Limit Pilot Scheme in Belfast City Centre	NI Government
May 2016	Northern Ireland Road Safety Strategy to 2020: Annual Statistical Report	NI Government

NI, Northern Ireland

### Analysis

All documents were imported to N-Vivo 12 software [37]. Our analysis followed the READ approach, which is recommended for documentary analysis in health policy research [16]. READ involves a four stage process to conduct document analysis for qualitative policy research: **R**ead materials, **E**xtract data, **A**nalyse data, **D**istill. The research team did not seek to develop themes from the data, which is common in qualitative research. Rather, we were interested in extracting information about evidence (whether, and how much, evidence was referred to throughout the deliberation processes) and the ways in which evidence was used (for example, to make the case for the policy, or in defence against opposition). This information was used to construct a chronological timeline of evidence use in each city. Documents relevant to Edinburgh and Belfast were analysed separately due to the differing contexts and the nature of the interventions (city-wide versus city centre). Data were extracted independently by two members of the research team (K.M. and M.K.), who then worked collaboratively to piece together a chronological narrative of the role that evidence played in decision-making in each of the cities.

## Results

The role of evidence in the decisions to implement large-scale 20 mph speed limits in Edinburgh and Belfast is presented as two case studies below.

### The Edinburgh narrative

Over many years, the national transport-related documents in Scotland consistently drew upon evidence about speed and safety. For example, in 2009 the Scottish Government published *Go Safe on Scotland’s Roads: It’s Everyone’s Responsibility: Scotland’s Road Safety Framework to 2020* [42]. This document included a specific chapter on evidence, in which the importance of evidence in informing every stage of policy-making and delivery was emphasized. The report highlighted a range of statistics related to injuries and deaths of pedestrians, cyclists and car users on Scotland’s roads.

In June 2010, the Scottish government launched the country’s first Cycling Action Plan for Scotland (CAPS), with an aspiration that, by 2020, 10% of all journeys taken in Scotland would be by bicycle [43]. The key evidence considered in the development of CAPS came from over 6000 qualitative responses to a public consultation on the barriers to increased cycle use and the measures people felt were required to get “more people cycling more often”. That qualitative evidence highlighted that reducing vehicle speeds would be a key factor in encouraging people to make the choice of walking or cycling. This was considered sufficient evidence by decision-makers to make the case, despite a lack of quantitative data on the relationship between vehicle speed and active travel choices.

In 2014, Transport Scotland (the national transport agency for Scotland) published the *Good Practice Guide on 20 mph Speed Restrictions* (Transport [50]). The *Good Practice Guide* noted that higher speeds lead to collisions that are more serious. It used data from the UK Department for Transport concerning pedestrian fatalities and speed as well as speed and the number of collisions, which drew in turn upon data from the European Transport Safety Council [19, 20]. It referred to much international evidence supporting these arguments (e.g. [54]). It noted that excessive speed is reported in 13% of all reported collisions and 20% of fatal collisions.

At the local authority level, there were references to evidence in many of Edinburgh Council’s own documents. In 2010, the council explored the relationship between speed and risk [8]. Echoing the national narrative, the evidence was interpreted to mean that risk increases slowly until impact speeds of about 30 mph. The council noted that even though the risk of a pedestrian fatality at 30 mph is “relatively low”, approximately half of pedestrian fatalities occur at that impact speed or below.

The Council document argued that vehicle speed was the most important single factor in the severity of road collisions, with the risk of fatal injury to pedestrians being more than eight times higher at 30 mph than 20 mph. It noted that the chance of survival halves between 30 mph and 40 mph. It also observed that streets with slower traffic are more attractive to residents, pedestrians, cyclists and children, and can improve the environment for business and social interaction. It was argued that cars travelling at 20 mph generate less noise. An emphasis was placed on the fact that a high proportion of pedestrian and cyclist casualties occur on the busiest streets in the inner areas of the city. Whilst it was noted that in many of these streets, average speeds were already relatively low, it was suggested that a 20 mph limit had the potential to help rebalance street use in favour of pedestrians and cyclists.

The Council then planned a pilot study of the implementation of lower speed restrictions in the south of the city. A pilot scheme would allow the Council to demonstrate the feasibility of implementing 20 mph speed limits at scale, as well as to collect before and after data to show the impact of the new 20 mph limit on speed, collisions and casualties. Therefore, in addition to “using” evidence, the Council committed to “generating” the evidence it felt was needed to convince people to support the policy.

A proposal for a 20 mph Speed Limit Pilot in South Edinburgh was submitted to the Transport Infrastructure and Environment Committee of the council on 21 September 2010, seeking approval for a large-scale pilot of a 20 mph speed limit in residential streets [7]. This document included many references to evidence including the effectiveness of 20 mph zones in parts of Edinburgh and the reported reductions in average vehicle speed and casualties following the introduction of 20 mph speed limits in Portsmouth. Reference was also made to the Active Travel Action Plan, which claimed that lower traffic speeds can help in encouraging walking and cycling [7]. At the Transport, Infrastructure and Environmental Committee on 2 August 2011, approval was given to introduce a 20 mph speed limit on a number of roads on the south side of the city [8].

The pilot scheme was launched on 23 March 2012. The evaluation report showed some contradictory findings. Average speeds before implementation were 22.8 mph, while after implementation speeds fell to 20.9 mph; an average fall of 1.9 mph. Four locations across the pilot saw slight increases in average vehicle speeds from the “before” to the “after” survey; four locations continued to have average speeds at or above 24 mph; and there was an overall increase in the number of vehicles on most streets from the “before” to the “after” period, although in no location was this deemed “notable” [10].

In reporting the attitudes of residents “*[t]he main benefits of the pilot, as viewed by residents, were (in priority order) safety for children walking about the area, safety for children to play in the street, better conditions for walking, less traffic incidents, and better cycling conditions”* [10]. The evaluation report stated that: *“The overall level of support for the 20 mph speed limit has increased from 68% ‘before’ to 79% ‘after’, while the proportion of respondents strongly supporting the 20 mph speed limit increased significantly from 14% ‘before’ to 37% ‘after’. Only 4% were opposed, from 6% ‘before’”* [10].

Whilst the evaluation findings were slightly mixed, the Council felt that having no evidence against the pilot was sufficient to take scaled-up action. The council claimed that the intervention encouraged a slower and safer environment and for journeys to be undertaken by environmentally friendly modes of walking and cycling [10]. The idea of a more liveable, cleaner, sustainable, healthier city was thus woven into the narrative.

A narrative originally about the specific intent to reduce collisions and casualties gradually shifted to the idea that 20 mph would contribute to a wider range of aspirations. By 2011, Edinburgh Council documents relating to the slower speed limit were arguing that 20 mph would contribute to people living longer healthier lives, free from crime and disorder, in well-designed, sustainable places with access to amenities and services [9]. The suggestion was that it would be easier to value and enjoy the built and natural environment and protect and enhance it for future generations. This in turn would reduce the local and global impact of consumption and production. No attempt was made to cite any evidence for these potential wider benefits of 20 mph speed limits. By 2014, the council had stopped referring to evidence on road safety, and instead were pursuing a more general public relations exercise to generate support for its plans [11].

Following almost 10 years of discussion, approval was granted in March 2015 for the roll-out of the city-wide 20 mph network [12]. The scheme would be introduced using a staged approach across six areas. This would allow comparisons to be made on factors that influence effectiveness including physical characteristics such as topography, junction density and the extent of parking available, as well as “human characteristics” such as relative affluence/deprivation, demographic distribution and car ownership.

### The Belfast narrative

There is a rich stream of transport-related documents emanating from the Northern Ireland Administration. In November 2002, the Northern Ireland Road Safety Strategy (2002–2012) was published to address rising trends in road traffic casualties [34]. The evidence used to inform the strategy came from a consultation that was distributed widely and sought input on: what would represent challenging yet realistic targets; the combination of existing and new measures that would be needed to achieve these; and, in particular, ideas about how best to reduce road casualties and to secure the commitment of road users to improving road safety. Responses were received from more than 70 organizations and individuals. The strategy stated an objective to improve road safety for pedestrians and other vulnerable road users. This would be achieved by influencing drivers to avoid excessive speed and to drive more responsibly, although it was not explicit as to how this would be achieved.

In April 2010 the Department for Infrastructure published a document entitled *Setting Local Speed Limits*, which presented a range of evidence [32]. It argued that 20 mph zones are very effective at reducing collisions and casualties. It noted that 20 mph may reduce overall average annual collision frequency by around 60%, and the number of collisions involving children may be reduced by up to two thirds. It observed that 20 mph zones help reduce traffic flow and reduce casualties by over a quarter (quoting [53]). They also, it noted, produced a shift towards more walking and cycling. The authors pointed out that signed-only 20 mph speed limits generally led to only small reductions in traffic speeds and are most appropriate for areas where vehicle speeds are already low [32].

The same document pointed out that there was clear evidence of the impact of reducing traffic speeds on collisions and casualties, as collision frequency is lower at slower speeds, and where crashes do occur, there is a lower risk of fatality at lower speeds. It noted that on urban roads with low average traffic speeds, any 1 mph reduction in average speed can reduce the collision frequency by around 6% (quoting [47]). It argued that the other benefits of 20 mph speed limit interventions include quality of life and community benefits, and encouragement of healthier and more sustainable transport modes such as walking and cycling, although no evidence was cited to support these claims. It suggested that there may also be environmental benefits, as generally driving more slowly at a steady pace will save fuel and reduce carbon dioxide emissions, unless an unnecessarily low gear is used.

Later documents made reference to the economic benefit of preventing collisions and casualties. For example, one document published in 2014 stated that the average value of preventing a collision is approximately £72 700 [49]. It continued that an approximate saving of this amount can be made every time a collision is prevented by means of road safety engineering.

A paper published in 2014, examining key trends in road traffic collisions in Northern Ireland, observed that over the previous 13 calendar years the number of people killed annually on Northern Ireland roads had reduced significantly [27]. However, it went on to observe that although the number of people killed had declined significantly over the previous decade, between 2005 and 2012, the number of casualties actually increased – because of an increase in slight injuries. This document reported that vulnerable road users (i.e. pedestrians, pedal cyclists and motor cyclists) represent just over one third of the total number of fatalities between 2008 and 2012 and this remained relatively constant for each year [15 out of 48 in 2012 (31%) compared with 36 out of 107 (34%) in 2008].

*The Northern Ireland Road Safety Strategy to 2020* contained a large amount of information and evidence, largely focussed on the number of people of all ages killed or seriously injured on the roads [35]. The same document also considered inequalities in child pedestrian casualties [35]. It reported that child pedestrian casualties (aged 0–15 years) were higher in more deprived areas and that this relationship was highly statistically significant, with a trend that was stronger for male pedestrians than for female pedestrians and for children than for adults. The authors noted that a child living in the most deprived area is almost five times more likely to be injured in a collision than a child living in a least deprived area.

The strategy contained a commitment to pilot schemes for signed only 20 mph limits. However, it took a further 5 years for this commitment to translate into tangible action. It is worth noting a distinction in the use of the term “pilot” in the two cities. In Edinburgh this term was used to describe a trial which, if successful, would be scaled up across the city. In Belfast, there was no small-scale trial, rather, they proceeded straight to the full-scale city centre intervention. The term pilot implied the scheme may have been removed if proven to be ineffective, although it cannot be known whether or not that would have been the case.

In Belfast, although the narrative was less all-embracing than Edinburgh, the proclaimed benefits of 20 mph broadened over time. The 2010 document on setting local speed limits presented a range of evidence [32]. In addition to reducing collisions and injuries, 20 mph restrictions were linked to overcoming social exclusion and strengthening rural communities, as well as aiding wider economic and environmental objectives, although no specific evidence was cited. It was noted that 20 mph speed limits might reduce overall average annual collision frequency by around 60%, and the number of collisions involving injury to children may be reduced by up to two thirds. Drawing upon published data, 20 mph “zones” (with traffic calming infrastructure) were observed to help reduce traffic flow, where research has shown a reduction in injuries by over a quarter as well as a modal shift towards more walking and cycling [53].

## Discussion

Policy narratives are the strategies used to influence beliefs and gain support for a particular course of action. Policy narratives are attempts to unite actors behind a common goal. They are not intended to directly modify behaviour, rather, they frame or create shifts in values and the ways in which problems are perceived, and may therefore be an important precursor to change [23]. Theory suggests that the success of such narratives is influenced by the receptiveness of the audience and/or how well the narrative aligns with their beliefs.

In Edinburgh, evidence demonstrating the impact of lower speed limits on collisions and casualties was used to gain attention on the potential benefits of 20 mph speed limits. However, the narrative shifted over time from a specific intent to reduce collisions and casualties, to 20 mph contributing to a wider range of aspirations, with no supporting scientific evidence about the wider benefits presented. For example, the council argued that their approach would contribute to people living longer healthier lives, in well-designed, sustainable places, free from crime and disorder. These interventions were also expected to contribute to enhancing the Council’s reputation for excellence. Whether this shift in narrative was deliberate, or emerged through the bureaucratic and political processes, or was simply absorbing wider social and cultural currents, is not possible to detect from the published documents. However, the shift may have helped to neutralize opposing discourses, which would have made it more difficult to introduce the new speed limit. Indeed, previous research has shown that very little opposition to the schemes was expressed on social media, which can be a frequently used platform for sharing disagreement with policy decisions [44].

In Edinburgh, we see a widening story; the discussions evolved into a tale of the common good [18, 41]. The political achievement was to turn the discourse of evidence into something non-partisan, with an appeal which went beyond the evidence and reached out to much more aspirational goals for the city. This might be because politicians were involved and had to develop an account that would be acceptable to a broad constituency, emphasizing very broad public health and community benefits. While the idea of traffic speed controls was hardly unthinkable – speed limits have been around for a long time – the idea of city-wide restrictions at a speed lower than 30 mph was a significant shift. What was achieved was to make the idea appear overwhelmingly appealing such that by the time it was enacted, it was to a significant degree mainstream and unexceptional – an example perhaps of changing perceptions of 20 mph such that it was within what has been referred to as the “Overton Window” [22].

The Belfast documents drew far more heavily on the scientific evidence on speed and risk than what was observed for Edinburgh. In Belfast, the narrative was less aspirational, although the proclaimed benefits of 20 mph speed limits also broadened over time. For example, 20 mph was linked to overcoming social exclusion and strengthening rural communities, as well as aiding wider economic and environmental objectives. Belfast was a smaller intervention, which was driven by the civil service, specifically those in transport. The whole effort has a much more administrative feel to it, which may be why the Belfast documents presented more evidence.

Building or honing the narrative in a variety of ways for different audiences and purposes is rather akin to what Galea called incremental gradualism [23], or Ogilvie et al. referred to as pragmatic pluralism [36]. Indeed, storytelling is even now embraced by epidemiologists who acknowledge its power for galvanizing action and building a bridge to evidence translation [14]. In the context of the Edinburgh and Belfast interventions, this means finding a way to make the policy change palatable, and perhaps even agreeable, among a wide range of stakeholders and the general public.

It is well recognized that scientific evidence, in isolation, is generally insufficient to achieve policy change. Policy-making is not a systematic step-by-step process but rather a fluid deliberative process [46]. This paper sought to understand the use of evidence in the deliberative processes of speed limit policies in two major UK cities. The policy-making processes in both cities required the clever and subtle use of policy narratives to drive change. Once the benefits of 20 mph were recognized, the next step for policy-makers was to establish how to get the interventions in place. Thus the question shifted from “is there evidence that we have a problem?” or “is there evidence that such interventions will work in the way we think they will?” to “how do we make this happen in practice?” In the case of Edinburgh, this was via a process of bringing various parties on board and appealing to the greater good of the city as a whole. In Belfast, it was done administratively through the civil service and thus there was little room for political contestation of evidence. That said, the Northern Ireland Executive’s policy “playbook” acknowledges the need to go beyond the evidence and consider implementation challenges from the outset of any policy-making journey [31].

These case studies show that evidence was not used to enact decisions but was used astutely to influence others to support the schemes, such that when implementation eventually occurred, it appears to have been regarded in the communities as largely unremarkable [55]. The changing narratives had framed the idea in such a way that it had broad acceptability [4, 21, 52].

It is worth noting that in both cities the 20 mph speed limits are still in place, and it is anticipated that they will remain a permanent feature. In Edinburgh discussions have begun about potentially reducing 40 mph streets to 30 mph [51]. Since the implementation of the Belfast intervention, 20 mph limits have been introduced outside 100 schools, and discussions are underway about widening the intervention further [33].

Evidence is a starting point, but a lot has to happen to bring interventions such as these to fruition. The cases of Edinburgh and Belfast are worth examining because they did successfully implement speed restrictions, and in Edinburgh in particular, there has been a marked decline in collisions, casualties and fatalities, and positive trends in Belfast for a smaller, city centre intervention, were also observed [25]. The implementing organizations drew upon the descriptive evidence about the effects of speed restriction. Nevertheless, on its own that evidence did not lead to the political position that allowed the local jurisdictions to act. The evidence had to go through a process of honing and shaping to provide the basis for action. It took rather different directions in the two cities – their tales are different – but in the end, the different tales fitted the local political contexts.

Some limitations of this work may be noted. Our analysis was confined to documentary evidence. Whilst policy documents provide a detailed account of evidence and proposed actions, they may provide only a partial picture of the types of evidence that were considered and how that evidence influenced decision-making. Secondly, documents often provide a specific storyline and may reflect what the relevant authorities wanted to convey about the process, rather than necessarily revealing the full truth about what happened in reality. Thirdly, whilst all documents were analysed independently by two researchers, those researchers inevitably bring their own pre-conceived ideas and biases to the process; it is possible that other researchers may have drawn different conclusions from the evidence. Finally, we sought to explore the discourse that emerged throughout the years of deliberation on the schemes, rather than to test a particular theory of policy-making. It is possible that the application of one or more theoretical frameworks may have yielded different or additional insights.

## Conclusions

In recent years, the UK cities of Belfast and Edinburgh introduced 20 mph speed limit interventions – city-wide in Edinburgh and in the city centre in Belfast. Tales, or narratives, about the use of evidence prior to the successful introduction of the slower speed restrictions were constructed in both places. The relationship between evidence and decision-making in Edinburgh and Belfast was neither simple nor linear. While organizations such as national governments may well be imbued with a culture that recognizes the importance of evidence, evidence still has to find its way into practice at a local level. One element in the process concerns the way evidence is actually used, not only to guide decision-making, but also in the political processes that precede and accompany formal decision-making.

These narratives are some way distant from the hard data points and *P*-values of primary evidence of purported effectiveness and are in themselves part of an interpretative process of that evidence. Nevertheless, we contend that the narratives – the tales or stories – were critically important in the way things evolved in the two cities, and without which there would have been no implementation, and probably a lot more push back from vested interests and communities than there was.

## Data Availability

Much of the data are publicly available. We have made it clear which documents are not publicly available.

## References

[1] Adams J, Hillier-Brown FC, Moores HJ, Lakes AA, Araujo-Soares V, White M, Summerbell C. Searching and synthesising ‘grey literature’ and ‘grey information’ in public health: critical reflections on three case studies. Syst Rev. 2016;5:164. 10.1186/s13643-016-0337-y. 27686611 10.1186/s13643-016-0337-y PMC5041336

[2] Brownson RC, Fielding JE, Maylahn CM. Evidence-based public health: a fundamental concept for public health practice. Ann Rev Public Health. 2009;30:175–201. 19296775 10.1146/annurev.publhealth.031308.100134

[3] Burstein P. The determinants of public policy: what matters ad how much? Policy Stud J. 2020;48(1):207–30. 10.1111/psj.12243

[4] Cairney P. Evidence-based best practice is more political than it looks: a case study of the ‘Scottish approach.’ Evid Policy. 2017;13(3):499–515. 10.1332/174426416X14609261565901

[5] Cairney P, Kippin S. Politics and policy making in the UK. Bristol: Bristol University Press; 2023.

[6] Charlesworth G. A history of the transport and road research laboratory 1933–1983. Aldershot: Gower; 1987.

[7] City of Edinburgh Council—Transport and Environment Committee. Proposal for 20mph speed limit pilot in South Edinburgh. Edinburgh: City of Edinburgh Council; 2010.

[8] City of Edinburgh Council—Transport and Environment Committee. 20mph speed limit pilot in South Edinburgh—Variation to traffic regulation order. Edinburgh: City of Edinburgh Council; 2011a.

[9] City of Edinburgh Council—Transport and Environment Committee. South Edinburgh 20mph limit pilot—Response to TRO consultation. Edinburgh: City of Edinburgh Council; 2011b

[10] City of Edinburgh Council—Transport and Environment Committee. South Central Edinburgh 20mph limit pilot evaluation. Edinburgh: City of Edinburgh Council; 2013.

[11] City of Edinburgh Council—Transport and Environment Committee. Delivering the Local Transport Strategy 2014–2019: 20mph speed limit roll out—consultation proposal. Edinburgh: City of Edinburgh Council; 2014.

[12] City of Edinburgh Council—Transport and Environment Committee. 20 for Edinburgh: 20mph network implementation. Edinburgh: City of Edinburgh Council; 2015.

[13] Cleland CL, McComb K, Kee F, Jepson R, Kelly M, Milton K, Nightingale G, Kelly P, Baker G, Craig N, Williams A, Hunter R. Effects of 20 mph interventions on a range of public health outcomes using the meta-narrative method. J Transp Health. 2020. 10.1016/j.jth.2019.100633. 10.1016/j.jth.2019.100633

[14] Cofiño R, Prieto M, Suárez O, Malecki K. The art of drawing numbers and stories in the air: epidemiology, information, emotion and action. Epidemiol Commun Health. 2014. 10.1136/jech-2014-203883. 10.1136/jech-2014-203883 25190819

[15] Crammond B, Carey G. What is policy and where do we look for it when we want to research it? Epidemiol Commun Health. 2016. 10.1136/jech-2016-207945. 10.1136/jech-2016-207945 27864323

[16] Dalglish SL, Khalid H, McMahon SA. Document analysis in health policy research: the READ approach. Health Policy Plan. 2020;35(10):1424–31. 10.1093/heapol/czaa064 PMC7886435 33175972

[17] Davies HTO, Nutley SM, Smith PC. What works? Evidence based policy and practices in public services. Bristol: Policy Press; 2000.

[18] Etzioni A. Reclaiming patriotism. Charlottesville: University of Virginia Press; 2019.

[19] European Transport Safety Council. Reducing traffic injuries resulting from excess and inappropriate speed. Brussels: European Transport Safety Council; 1995.

[20] European Transport Safety Council. Speed fact sheet 7: Setting appropriate, safe, and credible speed limits. Brussels: European Transport Safety Council; 2010.

[21] FrameWorks Institute. Framing and policy making—How does framing help advance our public policy goals? Washington: FrameWorks Institute; 2018.

[22] Galea S. Well. New York: Oxford University Press; 2019.

[23] Galea S. A radical vision, an incremental approach to achieving the aspirations of public health. Milbank Q. 2020. 10.1599/mqop.2020.1014.32333451 10.1599/mqop.2020.1014

[24] Government Office for Science. Realising our ambition through science: a review of government science capability. London: Government Office for Science; 2019.

[25] Jepson R, Baker G, Cleland C, Cope A, Craig N, Foster C, Hunter R, Kee F, Kelly MP, Kelly P, Milton K, Nightingale G, Turner K, Williams AJ, Woodcock J. Evaluation of 20mph speed limits in Edinburgh and Belfast on a range of public health outcomes: a mixed methods approach. London: National Institute of Health Research; 2022. 36173872

[26] Kelly MP. The relation between the social and the biological and COVID-19. Public Health. 2021;196:18–23. 34134011 10.1016/j.puhe.2021.05.003 PMC8114767

[27] McKibbin D. An overview of key road traffic collisions statistics in Northern Ireland. Belfast: Northern Ireland Assembly; 2014.

[28] Milton K, Kelly MP, Baker G, Cleland C, Cope A, Craig N, Hunter R, Foster C, Kee F, Kelly P, Nightingale G, Turner K, Williams AJ, Woodcock J, Jepson R. Use of natural experimental studies to evaluate 20mph speed limits in two major UK cities. J Transp Health. 2021;22: 101141. 10.1016/j.jth.2021.101141. 34603959 10.1016/j.jth.2021.101141 PMC8463832

[29] Milton K, Turner K, Baker G, Cleland CL, Foster C, Hunter RF, Jepson R, Kee F, Kelly P, Kelly MP. The processes of transport and public health policy change: 20mph speed limits in Edinburgh and Belfast. Case Stud Trans Policy. 2022;10:1855–61. 10.1016/j.cstp.2022.07.014

[30] National Institute for Health and Care Excellence. Physical activity: walking and cycling—Public health guideline [PH41]. London: National Institute for Health and Care Excellence; 2012.

[31] Northern Ireland Executive. A practical guide to policy making in Northern Ireland. Belfast: Northern Ireland Executive; 2016.

[32] Northern Ireland Government. Setting local speed limits in Northern Ireland. Belfast: Northern Ireland Government; 2010.

[33] Northern Ireland Government. *Mallon announces further roll out of 20mph speed limit schemes at 106 schools*; 2021. https://www.infrastructure-ni.gov.uk/news/mallon-announces-further-roll-out-20mph-speed-limit-schemes-106-schools. Accessed 6th Aug 2022.

[34] Northern Ireland Government. Northern Ireland Road Safety Strategy 2002–2012. Belfast: Northern Ireland Government; 2002.

[35] Northern Ireland Government. Northern Ireland’s Road Safety Strategy to 2020. Belfast: Northern Ireland Government; 2011.

[36] Ogilvie D, Bauman A, Foley L, Guell C, Humphreys D, Panter J. Making sense of the evidence in population health intervention research: building a dry-stone wall. BMJ Glob Health. 2020;5: e004017. 10.1136/bmjgh-2020-004017. 33298470 10.1136/bmjgh-2020-004017 PMC7733100

[37] QSR International Pty Ltd. NVivo (Version 12); 2018. https://www.qsrinternational.com/nvivo-qualitative-data-analysis-software/home.

[38] Reynolds JP, Stautz K, Pilling M, van der Linden S, Marteau TM. Communicating the effectiveness and ineffectiveness of government policies and their impact on public support: a systematic review with meta-analysis. R Soc Open Sci. 2020;7: 190522. 32218927 10.1098/rsos.190522 PMC7029938

[39] Sackett DL, Haynes RB, Guyatt GH, Tugwell P. Clinical epidemiology: a basic science for clinical medicine. Boston: Little Brown and Co; 1991.

[40] Sackett DL, Rosenberg WMC, Gray JAM, Haynes RB, Richardson WS. Evidence based medicine: what it is and what it isn’t. BMJ. 1996;312:71. 8555924 10.1136/bmj.312.7023.71 PMC2349778

[41] Sandel MJ. The tyranny of merit: What became of the common good? New York: Farrar, Strauss and Giroux; 2020.

[42] Scottish Government. Go safe on Scotland’s roads- it’s everyone’s responsibility: Scotland’s road safety framework to 2020. Edinburgh: Scottish Government; 2009.

[43] Scottish Government. Cycling Action Plan for Scotland (CAPS). Edinburgh: Scottish Government; 2010

[44] Semwal T, Milton K, Jepson R, Kelly M. Tweeting about twenty: analysing interest, public sentiments and opinion about 20mph speed restrictions in Edinburgh and Belfast using Twitter data. BMC Public Health. 2021. 10.1186/s12889-021-12084-x. 34836540 10.1186/s12889-021-12084-x PMC8626972

[45] Sense about Science. Transparency of evidence: an assessment of government policy proposals May 2015 to May 2016. London: Sense about Science; 2016.

[46] Smith K. Beyond evidence based policy in public health: the interplay of ideas. Basingstoke: Palgrave Macmillan; 2013.

[47] Taylor MC, Lynam DA, Baruya A. The effects of drivers’ speed on the frequency of road accidents. Crowthorne: Transport Research Laboratory; 2000.

[48] The Evidence-Based Medicine Working Group. Evidence-based medicine: a new approach to teaching the practice of medicine. J Am Med Assoc. 1992;268(17):2420–5. 10.1001/jama.1992.03490170092032 1404801

[49] Transport Northern Ireland. Road safety engineering procedures. Belfast: Transport Northern Ireland; 2014.

[50] Transport Scotland. Good practice guide on 20mph speed restrictions. Edinburgh: Transport Scotland; 2014.

[51] Turvill D. Edinburgh Council set to lower speed limit on 22 roads across the city; 2022. https://www.edinburghlive.co.uk/news/edinburgh-news/edinburgh-council-set-lower-speed-22885636. Accessed 6th Aug 2022.

[52] Tversky A, Kahneman D. The framing of decisions and the psychology of choice. Science. 1981;211:453–8. 7455683 10.1126/science.7455683

[53] Webster DC, Mackie A. TRL project report 215—Review of traffic calming schemes in 20mph zones. Crowthorne: Transport Research Laboratory; 1996.

[54] World Health Organization. World report on road traffic injury prevention. Geneva: World Health Organization; 2004.

[55] Yanovitzky I, Weber M. Analysing use of evidence in public policymaking processes: a theory-grounded content analysis methodology. Evidence & Policy. 2020;16(1):65–82. 10.1332/174426418X15378680726175

[56] Yin RK. Case study research and applications: design and methods. 6th ed. London: Sage; 2018.

